# Tumour microenvironment-responsive lipoic acid nanoparticles for targeted delivery of docetaxel to lung cancer

**DOI:** 10.1038/srep36281

**Published:** 2016-11-02

**Authors:** Fenfen Gu, Chuling Hu, Zhongguang Tai, Chong Yao, Jing Tian, Lijuan Zhang, Qingming Xia, Chunai Gong, Yuan Gao, Shen Gao

**Affiliations:** 1 Department of Pharmaceutics, Changhai Hospital, Second Military Medical University, Shanghai, 200433, China; 2 Department of Clinical Pharmacy and Pharmaceutical Management, School of Pharmacy, Fudan University, Shanghai, 201203, China

## Abstract

In the present study, we developed a novel type of reduction-sensitive nanoparticles (NPs) for docetaxel (DTX) delivery based on cross-linked lipoic acid NPs (LANPs). The physicochemical properties, cellular uptake and *in vitro* cytotoxicity of DTX loaded LANPs (DTX-LANPs) on A549 cells were investigated. Furthermore, the *in vivo* distribution and *in vivo* efficacy of DTX-LANPs was evaluated. The results showed that DTX-LANPs had a particle size of 110 nm and a negative zeta potential of −35 mv with excellent colloidal stability. LANPs efficiently encapsulated DTX with a high drug loading of 4.51% ± 0.49% and showed remarkable reduction-sensitive drug release *in vitro*. Cellular uptake experiments demonstrated that LANPs significantly increased intracellular DTX uptake by about 10 fold as compared with free DTX. The cytotoxicity of DTX-LANPs showed significantly higher potency in inhibiting A549 cell growth than free DTX, while blank LANPs had a good biocompatibility. In addition, *in vivo* experiments demonstrated that DTX-LANPs could enhance tumour targeting and anti-tumour efficacy with low systemic toxicity. In conclusion, LANPs may prove to be a potential tumour microenvironment-responsive delivery system for cancer treatment, with the potential for commercialization due to the simple component, controllable synthesis, stability and economy.

Chemotherapy remains the primary mode of treatment for cancer, a major threat to human health and life. However, conventional chemotherapies are highly nonspecific to cancerous cells and may produce substantial risks and adverse effects such as systemic toxicity^1^,^2^,^3^. Nano-drug delivery systems have shown a great potential to overcome such limitations to cancer chemotherapy^4^,^5^,^6^,^7^,^8^, and nanoparticles (NPs) have garnered much attention due to their outstanding capacity for active and passive targeting of drugs specifically into cancer cells^9^,^10^. To be a safe and effective drug delivery system, NPs should be stable enough for long circulation without causing premature drug release into the blood circulation^11^,^12^. Meanwhile, NPs should release the drug inside target tumour cells rapidly and completely^13^,^14^,^15^. As we all know, the tumour microenvironment is different from the microenvironment of normal cells, as the concentration of glutathione and other reducing substances in tumour cells is seven times as high as that in normal cells and is five thousand times as high as that outside normal cells^16^. Kim *et al*.^17^ reported that the disulfide bond could be cleaved rapidly via thiol-disulfide exchange reactions with intracellular reducing molecules, especially glutathione. Recent developments in reversibly cross-linked disulphide micelles have shown high stability in the circulation and effective drug release into tumour cells^18^,^19^. Our previous studies^20^,^21^,^22^ also demonstrated that disulphide-cross-linked micelles were capable of effectively carrying siRNA to target cells. In addition, other studies^23^,^24^,^25^,^26^ also reported that some disulphide micelles comprised lipoic acid conjugates could be used as vectors for drug delivery.

Lipoic acid is a natural antioxidant and has been applied to the treatment of Alzheimer’s disease and diabetes^27^,^28^. It is almost atoxic and listed as a healthcare product in Europe and the United States of America. Zhong *et al*.^29^ reported that the disulphide bond of the lipoic ring would rupture to form a poly-disulphide under a catalytic amount of cysteine and this rupture occurred in the cytoplasm of tumour cells rich in glutathione and other reducing substances. Disulphide cross-linked polymers can swiftly degrade in this setting, leading to a rapid drug release into the cytoplasm. Based on this principle, we demonstrated that cross-linked lipoic acid could be used as a novel high polymer material. Compared with actually high polymer materials such as polylactide-co-glycolide (PLGA), this novel material had stable “cross-linking scherm” that could prevent premature drug release and ensure long stability of NPs in circulation and break rapidly in response to the tumor microenvironment to release the drug totally. In addition, this novel material is easily synthesized, nontoxic, and biodegradable, thus will be suitable for commercialization.

## Results

### Synthesis, properties and characterization assay

**Figure 1:**
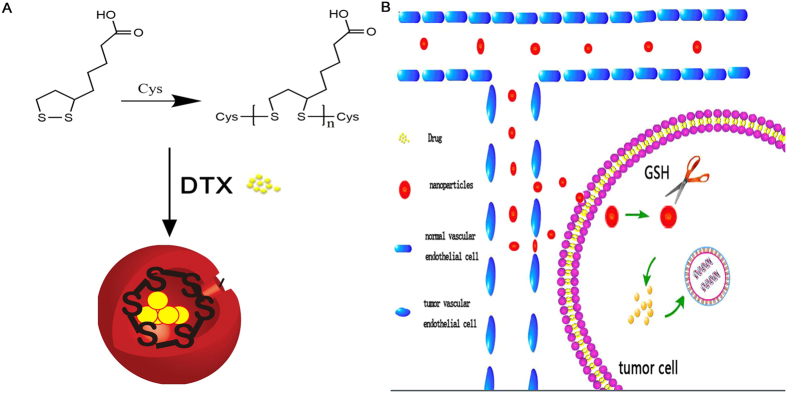
The syntheic scheme of cross-linked lipoic acid (**A**), and the action mechanism of LANPs (**B**).

**Figure 2:**
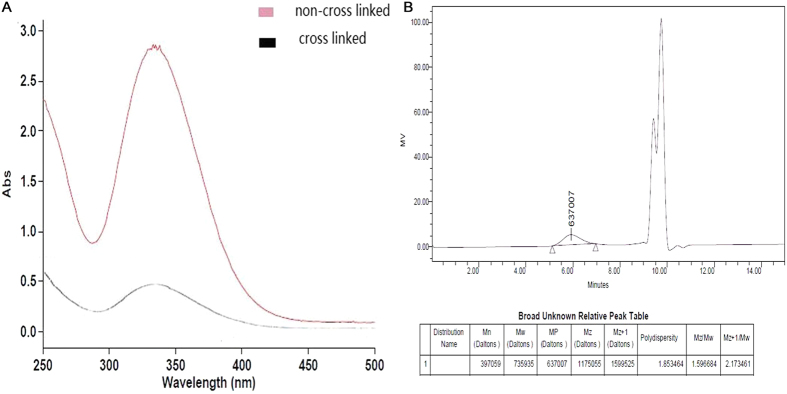
The graph of ultraviolet and visible absorption of cross-linked lipoic acid and non- cross-linked lipoic acid (**A**), and GPC of crosslinked lipoic acid (**B**).

The synthetic reaction of cross-linked lipoic acid and the working mechanism of LANPs are shown in Fig. 1. The verification of cross-linked lipoic acid by ultraviolet absorption and GPC is shown in Tables 1 and 2 and Fig. 2. It was found that lipoic acid contained a five-membered ring showing a specific absorption peak at 330 nm (Fig. 2A). When the reaction was triggered, the five-membered ring ruptured, followed by decrease of the specific absorption. As shown in Table 1, the ultraviolet absorption under different synthesis conditions has the lowest value in a 5:1 molar ratio of lipoic acid to cysteine at 8 h with the overall decrease of 91.33%. Additionally, with the cross-linking of lipoic acid, the molecular weight of the material was also the largest in a 5:1 molar ratio of lipoic acid to cysteine at 8 h, when the overall increase was about 99.72% (Table 2). Polydispersity index (PDI) of NPs represents the dispersibility and successful synthesis, and that PDI should be preferably less than 0.3. PDI of the NPs under different synthesis conditions was measured. As shown in Table 3, the PDI of all the NPs was less than 0.3 and the lowest PDI of NPs was in a 5:1 molar ratio of lipoic acid to cysteine at 8 h. These results indicated that the optimal synthesis condition was 8 h and a 5:1 molar ratio of lipoic acid to cysteine.

**Figure 3:**
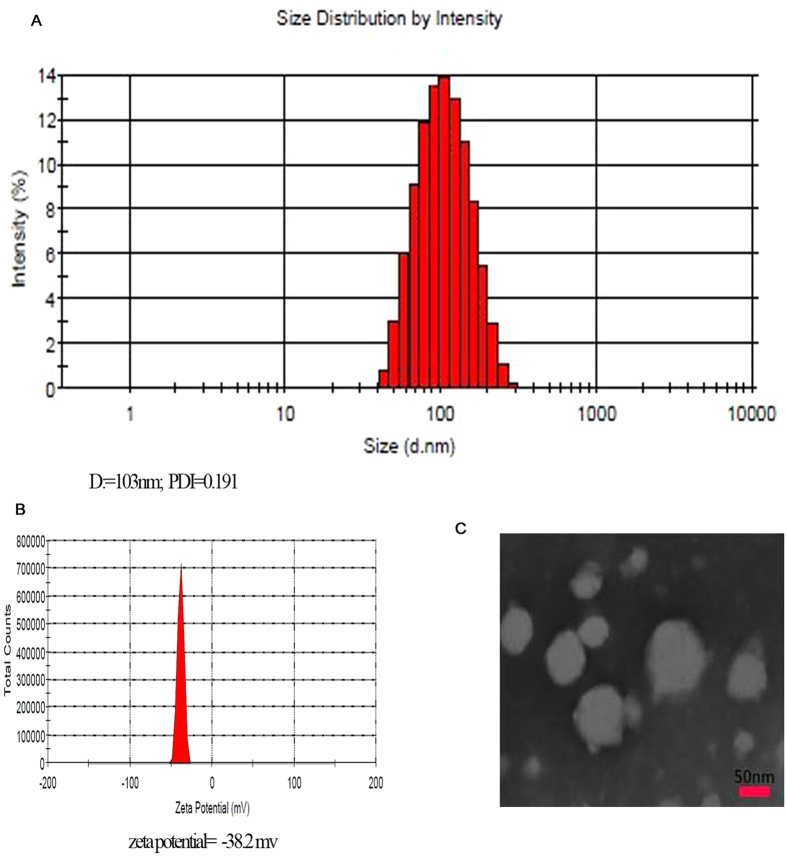
The size distribution and zeta potential of LANPs (**A,B**). The morphology of LANPs observed by TEM (**C**).

The size and zeta potential of LANPs in aqueous solution are shown in Fig. 3. NPs with small size (103.3 nm) and PDI (0.191) allowed for cellular uptake. The negative potential contributed to a high stability of LANPs in the blood. Transmission electron microscope (TEM) imaging displayed the clear spherical morphology of the LANPs and validated the size of the LANPs (Fig. 3C).

**Figure 4:**
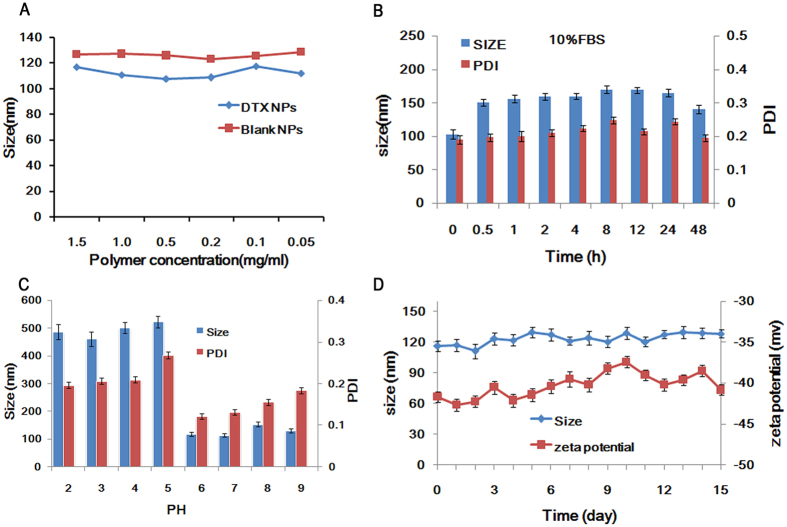
The effect of concentrations (**A**), 10% FBS (**B**), and pH (**C**) on the size and PDI of LANPs. The stability of LANPs was evaluated by size and zeta potential within 15 days in PBS (pH = 5.5 or 7.4) (D). Data are represented as mean  ± SD (n = 3).

The concentration and time stability were evaluated based on the size and zeta potential. The results showed that the particle size and zeta potential did not change significantly with the increased concentration (Fig. 4A). The particle size and PDI remained unchanged significantly during storage in 10% FBS at 4 °C (Fig. 4B). It was therefore concluded that LANPs were highly stable and resistant against aggregation even in 10% FBS, which may be a consequence of the ionized carboxyl group that prevented the NPs from binding with plasma proteins in the blood^30^. However, the size and PDI of LANPs was significantly affected by pH (Fig. 4C). When the pH dropped to 5, the size of the NPs suddenly became larger from about 100 nm to about 500 nm, and PDI increased by more than 0.2. The solution became cloudy, suggesting that aggregation may have occurred. These changes may be due to the protonation of the carboxyl on lipoic acid. The particle size and zeta potential did not change significantly with the lapse of time too (Fig. 4D).

**Figure 5:**
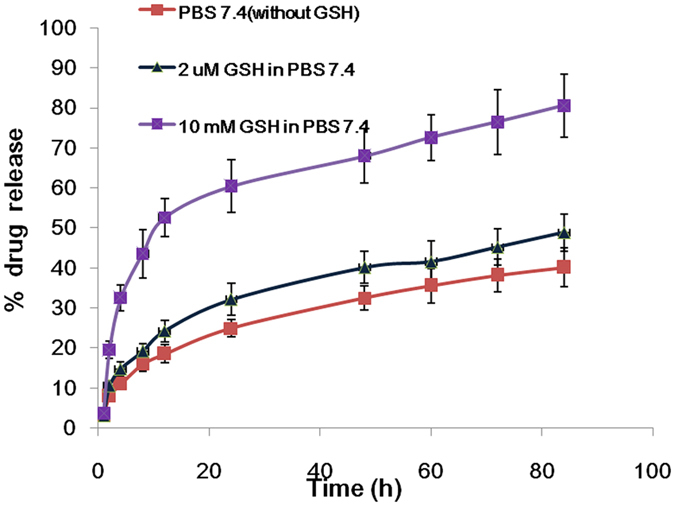
*In vitro* DTX release of DTX-LANPs in PBS containing 0.5% Tween-80 (B). Data are shown as mean ± SD (*n* = 3).

DTX-LANPs were successfully prepared with a high percentage of drug entrapment (EE) (90% ± 5.85%) and drug loading (DL) corresponding to 4.51% ± 0.49 (Table 4). The results indicated that LANPs were similar to PLGANPs in EE and DL. As DTX sediment appeared when the ratio increased to 5%, we did not increase the proportion. The *in vitro* drug release of DTX-LANPs was investigated at varying concentrations of GSH. As shown in Fig. 5, a rapid release took place in the first 24 h at all concentrations of GSH, and a slow release followed persistently during the subsequent 40 h. This biphasic release feature could allow for rapid tumour killing during the first phase and persistent release for continued killing during the second phase. The release rate varied with different concentrations of GSH. It was significantly faster at a high concentration of GSH than that at a low concentration, because the cross-linked disulphide bond would break in the presence of GSH.

The concentration of GSH in tumour cells is seven times that in normal cells, corresponding to an intracellular concentration 5000 times greater than the extracellular concentration^16^. DTX released in PBS without GSH was just about 20% in 24 h, indicating its long preventive effect of premature drug release in circulation, while DTX-PLGANPs released about 50% DTX in 24 h in PBS^31^,^32^. This property can be leveraged for controlling and facilitating drug release in tumour cells.

### Cytological evaluation

**Figure 6:**
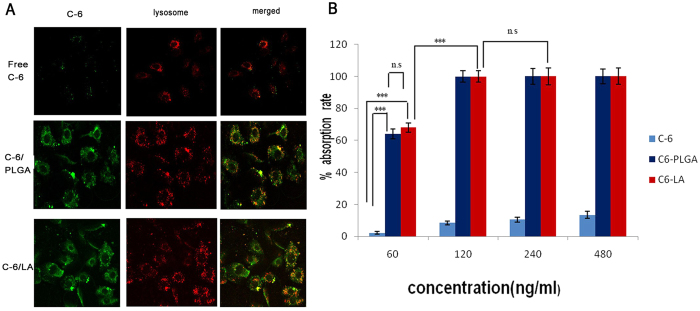
Cellular uptake of free coumarin 6 (C-6), coumarin-6 loaded LANPs (C-6/LANPs), and coumarin-6 loaded PLGANPs (C-6/PLGA) in A549 cells. CLAM images were observed after 4 h incubation with 60 ng/ml C-6 (**A**). C-6/NPs exhibited green, and the lysosome was stained by LysoTracker^®^ Red. The quantitative research of different concentrations of C-6 was tested by FCM in A549 cells. Data are shown as mean ± SD (n = 3). The comparison between two groups is analyzed by Independent Sample’s T test (ns. *p* > 0.05, ***p < 0.001).

Coumarin-6 loaded LANPs (C-6/LANPs) were used for quantifying the intracellular uptake by Flow Cytometry Method (FCM), and the endocytosis kinetics of the NPs was investigated by Confocal laser scanning microscope (CLSM) using coumarin-6 loaded PLGANPs (C-6/PLGANPs) as the positive control, and the free coumarin 6 (C-6) as the negative control (Fig. 6). As shown in Fig. 6A, the lysosome was stained by LysoTracker^®^ Red. The fluorescence in C-6/LANPs and C-6/PLGANPs groups was significantly higher than that in free C-6 group. The green fluorescence of the NPs distributed outside nucleus and partly in lysosomes, suggesting that the NPs could deliver DTX to the tumour cytoplasm to exert a tumouricidal effect and could escape lysosome partly. The intracellular uptake was quantified by flow cytometry. As shown in Fig. 6B, the mean fluorescence in C-6/LANPs and C-6/PLGANPs groups was significantly higher than that in free C-6 group (*p* < 0.001) for all concentrations. In addition, the mean fluorescence in C-6/LANPs was almost equal to that in C6/PLGANPs group (*p* > 0.05) for all concentrations. As the concentration was increased to greater than 120 ng/ml, the mean fluorescence in C-6/LANPs and C-6/PLGANPs groups plateaued, corresponding to about 100% uptake (*p* > 0.05). In summary, both qualitative and quantitative results suggested that the NPs allowed for excellent intracellular uptake.

**Figure 7:**
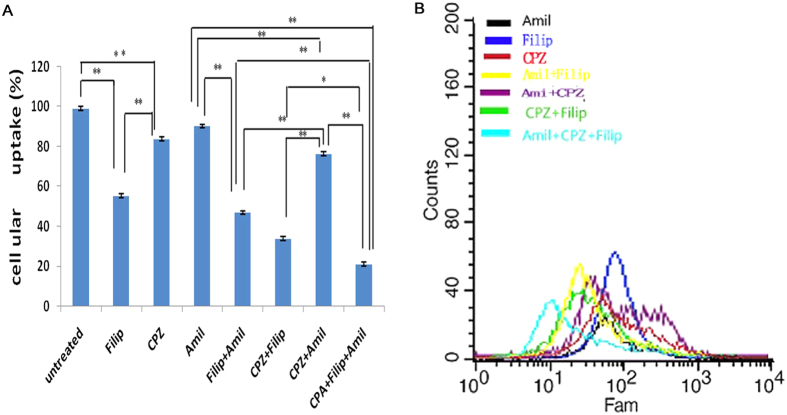
The endocytosis mechanism of LANPs. The flow cytometric data of LANPs uptake handled with different endocytosis inhibitors (**A**). Effects of different endocytic inhibitors of LANPs uptake on A549 cells (**B**). Data are shown as mean ± SD. (n = 3). *p < 0.05, **p < 0.01. Comparison between two groups is analyzed by Independent Sample’s T test.

In this study, several endocytic pathway inhibitors were employed to investigate the mechanism of trans-membrane uptake. Chlorpromazine (CPZ) is an inhibitor of clathrin-mediated endocytosis^33^, filipin (Filip) is an inhibitor of caveolae-mediated endocytosis^34^, and amiloride (Amil) can inhibit Na^+^/H^+^ exchange to prevent macropinocytosis^35^. As shown in Fig. 7, LANPs uptake was inhibited by CPZ (about 17%), Filip (about 45%), and Amil (about 9%). These findings demonstrated that caveolae-mediated endocytosis was the primary mechanism of the uptake of LANPs. Internalization, with clathrin-mediated endocytos, also played a role, while macropinocytosis only had an insignificant effect on the internalization of LANPs. In addition, the triple inhibition resulted in an about 21% decrease in NPs uptake, suggesting that other modes of internalization contributed to the uptake process. The mechanism of LANPs endocytosis is similar to that of PLGANPs^36^. Caveolae-mediated endocytosis constituted the major mechanism, probably due to the negative zeta potential of the NPs^37^.

**Figure 8:**
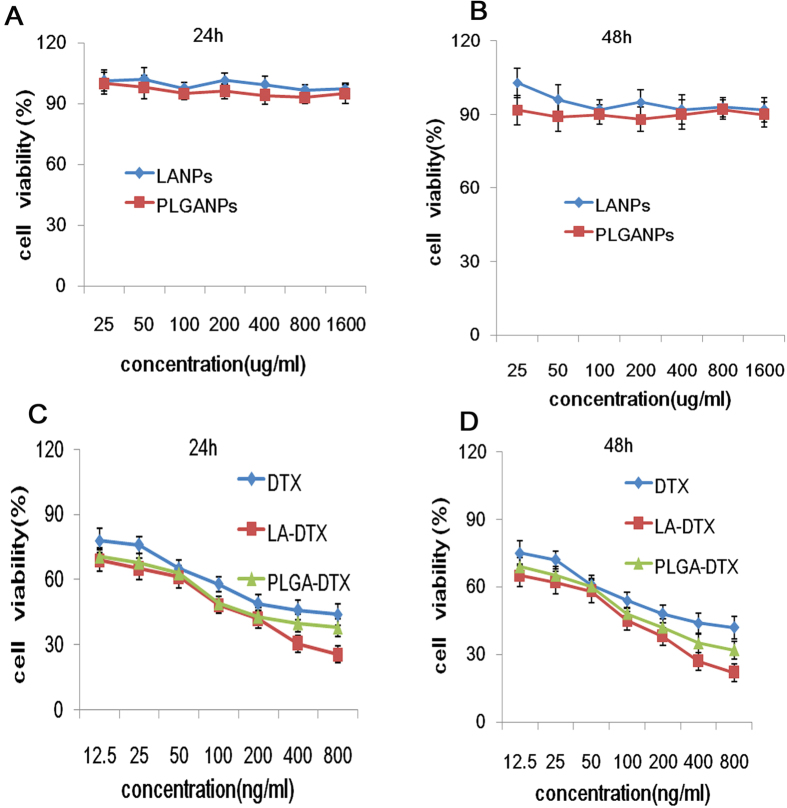
*In vitro* cytotoxicity of the NPs in A549cells at 24 and 48 h. The cytotoxicity of blank LANPs and PLGANPs (**A,B**), and free DTX-loaded LANPs (DTX-LANPs) and DTX-loaded PLGANPs (DTX-PLGANPAs) (**C,D**). Data are shown as mean ± SD (*n* = 3).

*In vitro* cytotoxicities of blank NPs and DTX loaded NPs evaluated by CCK-8 assay were shown in Fig. 8. As shown in Fig. 8A,B, the blank LANPs and PLGA-NPs showed almost no cytotoxicity in a series of concentrations up to 500 μg/mL. The cytotoxic effect at 24 h was almost similar to that at 48 h. Further, the cytotoxicity of DTX, DTX-PLGANPs and DTX-LANPs increased in a concentration-dependent manner (Fig. 8C,D). As shown in Table 5, the median lethal dose (IC_50_) of DTX-LANPs was significantly lower than that of DTX-PLGANPs (79.62 ± 1.98 ng/ml *vs.* 135.61 ± 3.12 ng/ml, *p* < 0.01), and these IC_50_ values were by far significantly lower than that of free DTX (277.84 ± 6.95) (*p* < 0.01), suggesting that the anti-tumour efficiency of DTX-LANPs was higher than that of DTX-PLGANPs. This may be due to the specific “cross-linking scherm” that broke in tumour cells to release the drug rapidly and totally. With the acting time of the NPs prolonging, the IC_50_ value decreased in all groups.

**Figure 9:**
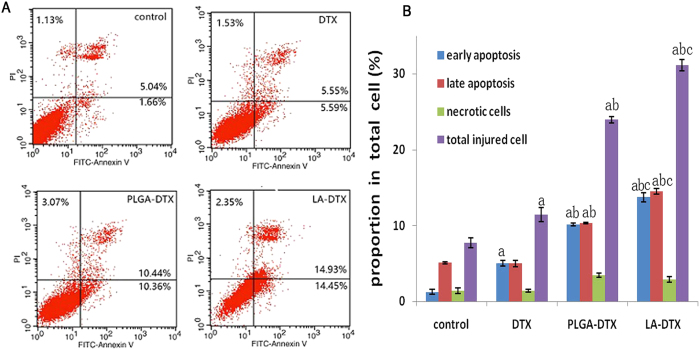
Cell apoptosis of A549 cells treated with free DTX, DTX-LANPs and DTX-PLGANPs at a DTX concentration of 10 ng/ml for 48 h. Flow cytometry data of different treatment groups (**A**). Quantitative analysis of cells apoptosis (**B**). a, *p* < 0.0 *vs*. control; b, *p* < 0.01 *vs.* DTX; c, *p* < 0.05 *vs.* DTX-PLGANPs (*n* = 3).

**Figure 10:**
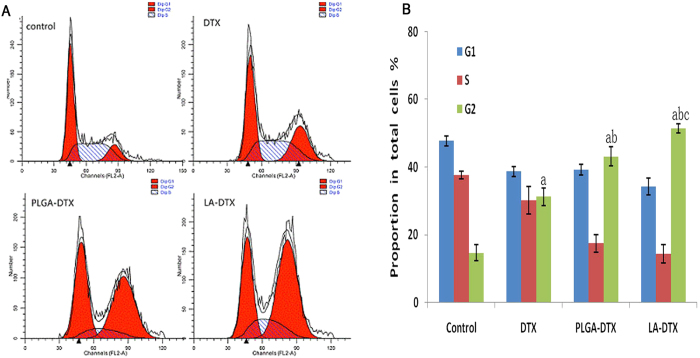
Cell cycle of A549 cells after treatment at a DTX concentration of 10 ng/ml with free DTX, DTX-LANPs and DTX-PLGANPs for 48 h. Flow cytometry data of different groups (**A**). Quantitative analysis of cell cycle (**B**). a, *p* < 0.0 *vs.* control; b, *p* < 0.01 *vs*. DTX; c, *p* < 0.05 *vs.* DTX-PLGANPs (*n* = 3).

As we know that cell cycle and apoptosis can prompt the anti-tumour mechanism.Cells were treated for 48 h with free DTX, DTX-PLGANPs, or DTX-LANPs at concentrations corresponding to 10 ng/mL DTX. As shown in Fig. 9A,B, the percentage of apoptotic and necrotic cells in the control group was negligible (<8%). However, the percentage of apoptotic cells in DTX, DTX-LANPs and DTX-PLGANPs groups underwent significant changes. The proportion of apoptotic cells in DTX-LANPs group was higher than that in DTX-PLGANPs or free DTX group. As shown in Fig. 10A,B, most cells in the control group distributed in G1 and S phases. However, cell cycle distribution underwent significant changes in all three treatment groups, where the percentage of cells in S phase was decreased, and the percentage of cells in G2 phase was increased. It is common knowledge that enzymes are compounds in S phase and RNAs and proteins are compounds in G2 phase. These changes in the percentage of cells in S phase and G2 phase suggest that cells passed S phase but mostly stagnated in G2 phase when they were treated with the drugs. This phenomenon was most evident in DTX-LANPs treatment group, and more evident in DTX-PLGANPs treatment group than that in free DTX treatment group. These results indicate that DTX-LANPs induced cell apoptosis most effectively as compared with DTX-PLGANPs and free DTX.

### *In vivo* anti-tumour assay

**Figure 11:**
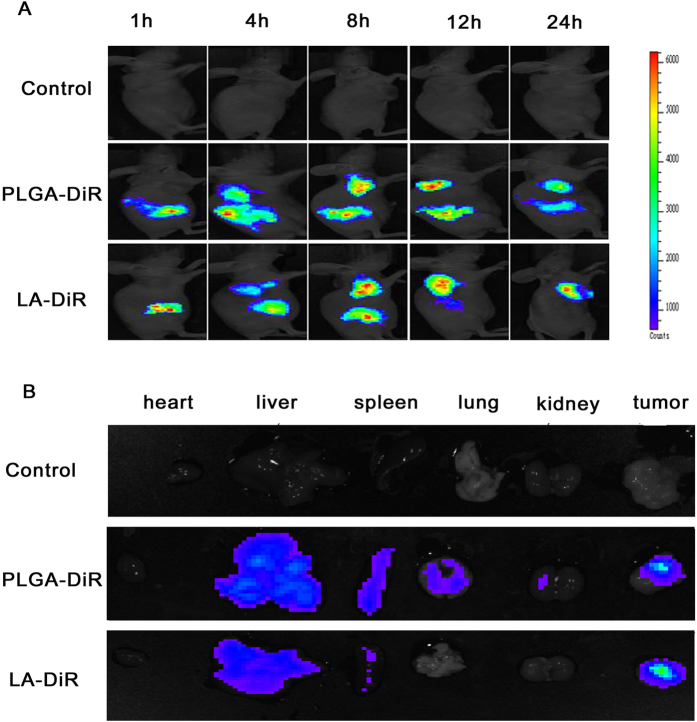
*In vivo* images of A549 transplantation tumour-bearing nude mice after injection with PBS, DiR loaded LANPs (LA-DiR) or DiR loaded (PLGA-DiR) through the tail vein. The images of flourescence distribution in the whole body at different time points (**A**). The images of excised organs and tumors *ex vivo* after 24-h injection (**B**).

NPs can passively target tumours because of the permeability retention effect (EPR) caused by leakage of blood vessels in tumours due to unregulated secretion of ngiogenic factors and decreased lymphatic drainage^38^. The abnormal vasculature decreases the efficient exchange of moleculesin to the bloodstream, thereby allowing for the accumulation and retention of NPs. The retention time is long enough to facilitate the uptake of the NPs by cancer cells via pinocytosis or to be exploited by the NPs, which use the retention time for self-disintegration and release of its contents in tumour cells and surrounding tissues^39^. In this study, DiR was used as the fluorescence signal to investigate NPs distribution in A549 tumour-bearing mice. Real-time images are shown in Fig. 11A. One hour after injection via the tail vein, DiR signals were found to accumulate in the liver, with a signal also appearing in the tumour as time progressed. Tumour DiR signals in the animals treated with the LANPs were stronger than those in the animals treated with PLGANPs at the same time point. The DiR signals reached the maximum at 8 h in the animals treated with LANPs, and at 12 h in the animals treated with PLGANPs, and then decreased gradually. Twenty-four hours after injection, the organs and tumours were harvested, and fluorescent images were collected (Fig. 11B). As shown by the images, the PLGANPs primarily accumulated in the liver, spleen, lungs, and tumour, while the LANPs primarily accumulated only in the liver and tumour, which may be due to the amphipathic nature of lipoic acid. The DiR signals in the tumours from the animals treated with LANPs were stronger than those from the PLGANPs treated animals, probably due to the stable “cross-linking scherm” that prevented premature DiR release and ensured that more DiR was delivered to the tumour. These results suggest that compared with the PLGANPs, the LANPs enhanced the tumour targeting property and produced fewer systemic adverse effects in the spleen and lung.

**Figure 12:**
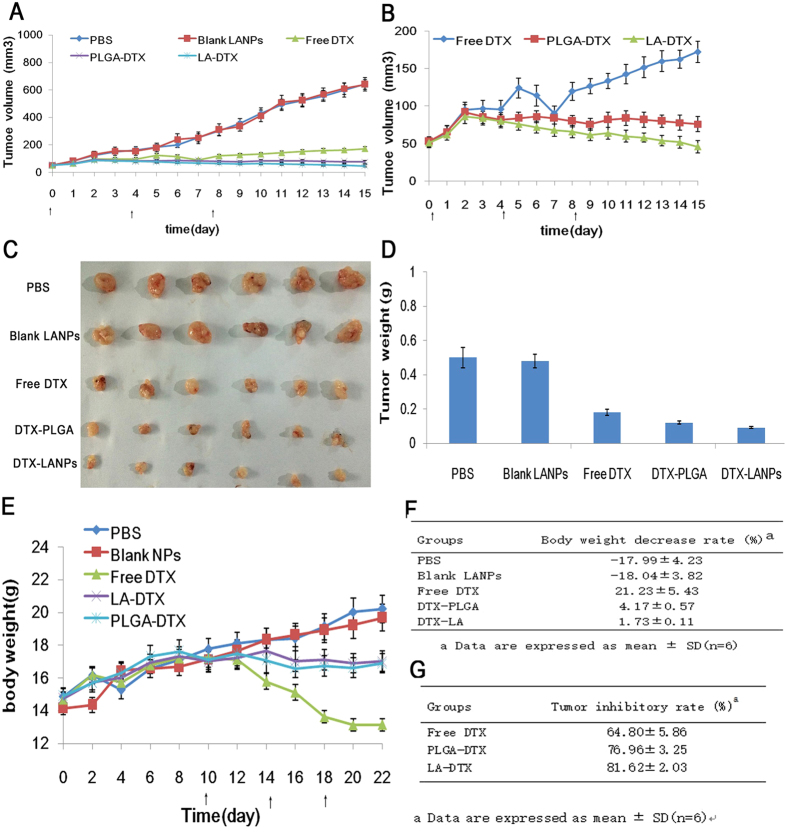
The anti-tumor efficiency *in vivo* in different treatment groups in A549 bearing nude mice. The change of tumor volume indicated tumor growth curves (**A**). The enlarged exhibition of tumor growth in free DTX, DTX-LANPs and DTX-PLGANPs treatment groups (**B**). The images of excised tumors (**C**). The tumor weighed at the end of test (**D**). The change of body weight of A549 bearing nude mice after treatment (**E**). The decrease rate of body weight was measured at the end of the test, “-” indicating body weight increase (**F**). The tumor inhibitory rate measured with excised tumor weight.

**Figure 13:**
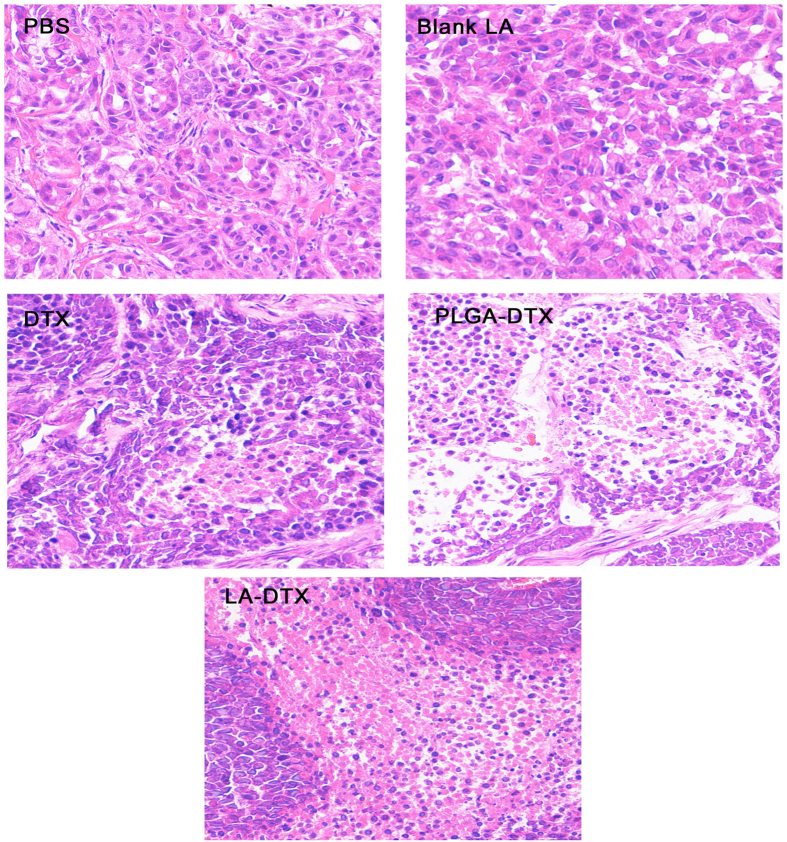
H&E stained tumors from A549 tumor-bearing nude mice treated with PBS, blank LANPs, DTX, DTX-PLGANPs or DTX-LANPs. The images were observed with a Leica microscope at 200X magnification.

The anti-tumour efficacy of the NPs was evaluated in A549 tumour-bearing mice. As shown in Fig. 12A, the PBS and blank LANPs had almost no anti-tumour effect (*p* > 0.05). The NPs showed a stronger anti-tumour effect than free DTX (*p* < 0.01), and the DTX-LANPs showed enhanced anti-tumour efficacy as compared with DTX-PLGANPs (*p* < 0.05) (Fig. 12B). The tumours were excised from the sacrificed animals and weighed (Fig. 12C,D). The DTX-LANPs exhibited higher anti-tumour efficacy than both DTX-PLGANPs (*p* < 0.05) and free DTX (*p* < 0.01) (Fig. 12G). H&E staining of a representative DTX-LANPs-treated tumour revealed widespread necrosis, while the tumour margins from the animals treated with PBS or the blank LANPs showed almost no change (Fig. 13). The safety of free DTX and DTX-NPs was assessed by measuring the changes in body weight (Fig. 12E). It was found that the body weight of the animals treated with PBS or blank LANPs did not decrease significantly, indicating that the blank LANPs had almost no toxicity. However, the body weight of the free DTX group decreased appreciably. Additionally, the body weight of the DTX-LANPs and DTX-PLGANPs groups was significantly decreased. The rate of weight loss in free DTX group was significantly higher than that in DTX-LANPs or DTX-PLGANPs groups (*p* < 0.01). Finally, the body weight decrease rate in DTX-LANPs group was lower than that in DTX-PLGANPs group (*p* < 0.05) (Fig. 12F). These results suggest that the DTX-LANPs had a great anti-tumour capacity against A549 xenograft tumours with minimal toxicity.

## Discussion

PLGA is one of the most extensively studied drug carriers for biomedical applications^40^. However, PLGA is known to be extremely hydrophobic and requires more than one year for complete degradation, which is too slow to satisfy the demands to be used for drug delivery^37^. In the present study, we designed amphipathic (lipoic acid) NPs at an optimal degree of disulphide cross-linking to overcome the drawbacks of PLGANPs.

The size of these NPs was smaller than 150 nm, which helps avoid their clearance in the reticuloendothelial system^41^. Their negative zeta potential was about 30 mv, probably owing to anionization of the carboxyl group of lipoic acid. The stability of LANPs was much better than that of PLGANPs, probably due to the ionized carboxyl group that prevents NPs from binding with plasma proteins in the blood^42^.

Various modes of NPs endocytosis could occur, including macropinocytosis, clathrin-mediated endocytosis and caveolae-mediated endocytosis^43^,^44^. The endocytic pathways vary with the size^45^, shape^46^, zeta potential^47^,^48^,^49^,^50^,^51^, coating^40^,^52^,^53^, concentration^54^ and fate of the internalized material^55^. The endocytic pathway of NPs affects their cellular destination, retention time and efficacy. Clathrin-mediated endocytosis exposes NPs to a hostile lysosomal environment, ultimately resulting in their degradation. NPs are internalized by caveolae-mediated endocytosis. However, circumvent lysosomal degrades and arrives in the endoplasmic reticulum, which proves to be effective for intracellular therapeutic delivery^56^. In the present study, we found that caveolae-mediated endocytosis was the major pathway of LANPs, and some LANPs enter the cells by clathrin-mediated pathway resulting in partly NPs distributed in lysosome. We chose DTX as the model drug. Release of the drug exhibited a GSH-sensitive property, which may reduce the adverse effects of DTX chemotherapy, because these LANPs can trigger tumour-specific release, thus reducing the DTX level outside tumour cells and in healthy cells. In conclusion, this “cross-linking scherm” prevented premature drug release, ensured long stability of NPs in circulation, and trigged tumour microenvironment responsive release. LANPs may prove to be an effective drug carrier for the treatment of cancer with improved efficacy and minimal adverse effects. In addition, these novel materials are easily synthesized, atoxic and biodegradable, thus suitable for commercialization.

## Materials and Methods

### Materials

Material used in this experiment were lipoic acid (Huayu Pharmaceutical Co., Ltd., Wuxi China); cysteine hydrochloride, sodium cholate, methanol and docetaxel (Sangon Biotech, Shanghai, China); PLGA (50:50, Mw35000, Lack Siomaterials, US); chlorpromazine hydrochloride and amiloride hydrochloride (Sigma-Aldrich, (St. Louis, MO, US); 4′,6-diamidino-2-phenylindole (DAPI) and Filipin III (Cayman Chemical, Michigan, US); 1,1-Dioctadecyl −3,3,30,30 – tetramethyl indotricarbocyanineiodide (DiR) (Biotium, CA, US); Apoptosis Assay Kit and Cell Cycle and Annexin V-FIT Kit (Becton Dickinson and Company, US); zeocin and puromycin (Invitrogen, Carlsbad, CA, US); RPMI-1640, foetalbovine serum (FBS), PBS and trypsin (Life Technologies, Grand Island, US).

Female nude mice aged 5 weeks and weighing about 16 g were purchased from the Experimental Animal Center of the Chinese Academy of Sciences (Shanghai, China). All animals were raised at the Second Military Medical University (Shanghai, China).

All animal experiments were performed in accordance with the protocols evaluated and approved by the ethics committee of the Second Military Medical University.

### Cell Culture

A549 cells (SBO Medical Biotechnology, Shanghai, China) were grown in RPMI-1640 supplemented with 10% FBS and antibiotics (100 U/mL penicillin andstreptomycin).

### Synthesis of cross-linked lipoic acid

To obtain cross-linked lipoic acid with varying degrees of cross-linking, 100 mg lipoic acid was mixed with different molar ratios of L-cysteine hydrochloride and then dissolved in 1 ml anhydrous methanol. The mixture was stirred for different lengths of time at room temperature. Nitrogen was used to blow-dry anhydrous methanol, and the cross-linked lipoic acid were then washed twice with dilutd hydrochloric acid and three times with distilled water. The cross-linked lipoic acid was then freeze-dried using a vacuum freeze drier. The synthesis was authenticated by characterizations.

LANPs were prepared by ultrasonic emulsification/solution^18^. Briefly, cross-linked lipoic acid (100 mg) was dissolved in 1 ml chloroform (organic phase) and 4 ml (1%) sodium cholate (aqueous phase). The organic phase and aqueous phase were sonicated with a disintegrator at 400 W for 1 min. Then, the emulsion was dispersed into 10 ml pure water and stirred for approximately 6 h to remove chloroform. Sodium cholate was removed by ultrafiltration. Briefly, NPs were placed in Amicon Ultra −15 ml filter units (100,000 MWD Merck Millipore) and centrifuged for 5 min at 3000 r/min. DTX-LANPs and C-6/LANPs were prepared by the same method, except that the organic phase was replaced by 100 mg lipoic acidin 5 mg DTX or 2 mg C-6. Unloaded DTX and C-6 were also removed by ultra-filtration.

### Characterization of LANPs and DTX-loaded LANPs

The specific absorption peak at 330 nm of the five-membered ring in lipoic acid was detected by ultraviolet and visible spectrophotometry (UV 2450/2550, Shimadzu, Japan). The molecular weight of cross-linked lipoic acid was determined by Gel Permeation Chromatography (GPC; HLC-8220, TOSOHCorporation, Tokyo, Japan).

The particle size, PDI and zeta potential of the LANPs (1.5 mg/ml) were measured by a Zeta sizer Nano ZS (Malvern, Westborough, MA, USA). The morphology of the NPs was examined by transmission electron microscopy (TEM, TecnaiG220, FEI Company, Hillsboro, Oregon, USA). Briefly, one drop of LANPs solution was placed on a 200-mesh copper grid coated with a carbon film. The grid was dried before TEM examination.

The NPs were prepared into different concentrations of 0.05–1.5 mg/ml with a pH value of 2–9, and stored at 4 °C for 15 days to evaluate their stability over time. Then, the NPs were prepared with 10% bovine serum in PBS to prevent protein aggregation. The stability was investigated by measuring the PDI and zeta potential of the NPs.

The DL and EE of the DTX-LANPs were measured by high performance liquid chromatography (HPLC, Agilent1260) equipped with a reverse phase C-18 column (150 mm × 4.6 mm, 5 μm, C_18_ Agilent Technologies, CA, USA). The mobile phase consisted of acetonitrile and deionized water (60/40). A flow rate of 1 ml/min was used with a detection wavelength of 230 nm and an injection volume of 20 μl. Syringe filters (0.45 mm pore size, Millipore, USA) were used to filter all samples before HPLC analysis.Different ratios of DTX and LA were investigated.

DTX-LANPs (1 ml) were placed in a dialysis tube (14000 Da). The tube was immersed in 20 ml PBS (pH7.4) containing 0.5% (w/w) Tween-80 and different concentrations of GSH and then placed in a shaker at a rate of 100 rpm at 37 °C. 1 ml of this medium was gathered at preset time intervals (1, 2, 4, 8, 12, 24, 48, 60, 72 and 84 h) with the addition of isovolumetric dissolution medium to maintain a sink condition. In addition, syringe filters (0.45 mm pore size, Millipore, USA) were used to filter all collected media before HPLC analysis.

### *In vitro* cellular uptake of coumarin 6-loaded NPs

C-6 is a model fluorescent probe and widely used to replace lipid-soluble drugs. C-6/PLGANPs and C-6/LANPs were prepared as previously described^18^. The concentration of C-6 was measured by fluorescent spectrophotometry. C-6/NPs were used for quantitative and qualitative assessment of cellular uptake in A549 cells by flow cytometry and confocal laser scanning microscopy. Cells were seeded in a 12-well plate (2 × 10^5^ cells/well) and incubated for 24 h.

C-6/LANPs, C-6/PLGANPS, and free C-6 (C-6) were added to A549 cells at varying C-6 concentrations (60, 120, 240 and 480 ng/ml). Cells were incubated for 4 h, washed, trypsinized, centrifuged, re-suspended in PBS, and finally analysed on a FACScan flow cytometer (Becton Dickinson, San Jose, CA, USA). A549 cells were cultured on microscope slides in a 24-well plate (2 × 10^4^ cells/well) and incubated for 24 h. C-6/ LANPs, C-6/ PLGANPs, C-6 were added into A549 cells with a final C-6 concentration of 60 ng/ml. After 4-h incubation, the culture medium was removed. Cells were washed three times with PBS, fixed with 4% paraformaldehyde solution for 30 min, and then were washed with PBS three times. 7–8 μl of the fluorescence quenching agent with DAPI was added dropwise onto the glass slides, and the glass slides were mounted onto the microscope slides. The slides were analysed by scanning confocal laser microscopy (TCS-SP5, Leica, Germany).

### Evaluation of endocytosis

A549 cells were seeded in a 12-well plate (2 × 10^5^ cells/well) and incubated for 24 h. To explore the internalization mechanism of LANPs, A549 cells were incubated with various endocytic inhibitors, including filipin III (1 μg/mL), chlorpromazine (30 μM), and amiloride (30 μM) for 1 h. Then, cells were incubated with C-6/NPs at a concentration of 120 ng/ml for 4 h, washed, trypsinized, centrifuged, re-suspended in PBS, and finally analysed on a FACScan flow cytometer (Becton Dickinson, San Jose, CA, USA).

### *In vitro* cytotoxicity

Cytotoxicities of blank NPs and DTX loaded NPs were on A549 cells were performed using a cell counting kit-8 (CCK-8) assay^14^. Briefly, A549 cells were seeded in a 96-well plate (1 × 10^4^ cell/well) and incubated for 24 h. Then, a series of concentrations of free DTX, DTX-LANPs, DTX-PLGANPs, blank LANPs, or blank PLGANPs were added to fresh media. Cells were incubated for 24 and 48 h, washed with PBS three times, and added with fresh medium (100 ml per well, containing 10 μl CCK-8 solution per well). After additional 1.5-h incubation, the absorbance of each well was measured at 450 nm using a microplate reader (Thermo, IL, USA). Cell viability was calculated using a percentage relative to the absorbance of the untreated cells^20^.

### Cell cycle and apoptosis assays

A549 cells were seeded in a 12-well plate (1 × 10^5^ cells/well), incubated for 24 h, and then assigned to four groups (three wells per group): (1) PBS group; (2) DTX group (free DTX 25 ng/ml); (3) DTX-LANPs group (25 ng/ml); and (4) DTX-PLGANPs group (25 ng/ml). After 24-h treatment, cell cycle distribution was analysed with Cell Cycle and Apoptosis Analysis Kit (Beyotime, Haimen, China) according to the manufacturer’s protocol^32^.

### *In vivo* anti-tumour assay

For evaluation of *in vivo* tumour targeting and distribution of the NPs, the near-infrared dye DiR was loaded into LANPs and PLGANPs. DiR-NPs were prepared in the same way as that for preparation of C-6/NPs, except that C-6 was replaced by 10 mg DiR. Free DiR was removed by ultrafiltration (10000 mw, 3000 r, 5 min). The DiR concentration in the NPs was measured by UV-Vis spectrophotometry^32^.

The animal experiments were performed on a xenograft tumour model. Female BALB/c nude mice aged 4 weeks and weighing about 16 g were injected with 1.0 × 10^6^ A549 cells subcutaneously on the right side of back. Ten days after inoculation, the mice were randomly divided into three groups: (1) PBS group; (2) PLGA-DiR (50 μg/ml) group; and (3) LA-DiR (50 μg/ml) group. The mice were injected with 200 μl solution via the tail vein. At 2, 4, 8, 12 and 24 h after injection, the mice were anesthetized with isoflurane, and the images were recorded by a Xenogen IVIS-200 imaging system equipped with a CCD camera (PerkinElmer Inc., MA, USA) at a 640 nm excitation wavelength. The mean fluorescence intensity of the tumour site was calculated using Xenogen IVIS-200 Software^20^.

The anti-tumour efficacy of the NPs was determined on the xenograft tumor model. Female BALB/c nude mice aged 4 weeks and weighing about 16 g were injected with 1.0 × 10^6^ A549cells subcutaneously on the right side of back. Two weeks after tumour inoculation (tumour volume about 50 mm^3^), 30 mice were equally randomized to five groups: (1) PBS group; (2) blank LANPs group; (3) free DTX group; (4) DTX-PLGANPs group; and (5) DTX-LANPs group. All the mice were injected via the tail vein every four days for a total of three injections (10 mg/kg).The weight of the mice and the tumour volume were measured every day. The tumour volume was calculated by the following equation: V = 0.5 × length × width^2^. The body-mass decrease rate (BDR) was calculated using the equation: BDR = [(W_f_ − W_i_)/W_f_] × 100%, where W_i_ and W_f_ refer to the initial body weight before treatment and the final body weight after treatment, respectively. After 15-day treatment, the mice were sacrificed and the excised tumours were weighed.

The tumour inhibitory rate was calculated with the equation: TIR = [(W_c_ − W_e_)/W_c_] × 100%, where W_c_ and W_e_ refer to the tumour weight of the control group and experimental groups, respectively^27^. Tumour tissues were analysed by H&E staining.

### Statistical analysis

Data are shown as the mean ± SD. Comparisons between two groups were measured by Independent Sample’s T -test. A P-value < 0.05 was considered to be statistically significant.

## Additional Information

**How to cite this article**: Gu, F. *et al*. Tumour microenvironment-responsive lipoic acid nanoparticles for targeted delivery of docetaxel to lung cancer. *Sci. Rep.* **6**, 36281; doi: 10.1038/srep36281 (2016).

**Publisher’s note:** Springer Nature remains neutral with regard to jurisdictional claims in published maps and institutional affiliations.

## Figures and Tables

**Table 1:** The ultraviolet absorption value at 330 nm UV under different synthesis condition^a^.

Time (h)	1	2	4	8	12	24
Mole ratios of LA per Cys						
2.5	3.642	3.528	2.685	1.842	1.428	1.326
5	1.539	1.254	1.228	1.001	1.336	1.422
10	1.364	1.367	1.338	1.031	1.054	1.542
15	1.652	1.528	1.423	1.411	1.325	1.352

^a^The absorption of lipoic acid at the same concentration was 11.542.

**Table 2:** The Molecular Weight ^a^ (kDa) of cross-inked lipoic acid under different synthesis condition.

Time (h)	1	2	4	8	12	24
Mole ratios of LA per Cys						
2.5	387	413	459	594	568	569
5	547	604	613	736	589	570
10	576	576	580	704	657	542
15	512	548	553	584	570	571

^a^The Molecular Weight (kDa) obtained by gel permeation chromatography (GPC) analysis.

**Table 3:** PDI of nanoparticles under different synthesis condition.

Time (h)	1	2	4	8	12	24
Mole ratios of LA per Cys						
2.5	0.236	0.264	0.258	0.124	0.152	0.120
5	0.244	0.226	0.248	0.098	0.141	0.147
10	0.247	0.246	0.212	0.139	0.108	0.118
15	0.238	0.289	0.22	0.189	0.192	0.187

**Table 4:** Characterization of DTX-LANPs and DTX-PLGANPs.

DTX-LANPs	Size (nm)	Zeta potential (mv)	Encapsulation efficiency (%)	Drug loading (%)
0:100	103.3 ± 1.7	−36.3 ± 3.1		
2:100	107.6 ± 1.5	−35.6 ± 2.6	78.56 ± 2.21	1.58 ± 0.10
3:100	110.2 ± 2.3	−37.5 ± 3.2	81.33 ± 2.3	2.56 ± 0.18
4:100	108.4 ± 1.6	−38.5 ± 2.1	76.26 ± 4.13	3.12 ± 0.14
5:100	106.2 ± 1.5	−38.5 ± 1.5	90.00 ± 5.85	4.51 ± 0.49
DTX-PLGANPs (5:100)	120.3 ± 2.4	−28.4 ± 1.8	91.60 ± 5.74	4.78 ± 0.56

**Table 5:** The IC_50_ value in different treatment groups as measured by the formula of logarithmic curves.

Time	Free DTX	PLGA-DTX	LA-DTX
24 h	277.84 ± 6.95	135.61 ± 3.12	79.62 ± 1.98
48 h	210.84 ± 4.91	94.11 ± 1.88	59.39 ± 1.18
